# Reliability and validity of multicentre surveillance of surgical site infections after colorectal surgery

**DOI:** 10.1186/s13756-022-01050-w

**Published:** 2022-01-21

**Authors:** Janneke D. M. Verberk, Stephanie M. van Rooden, David J. Hetem, Herman F. Wunderink, Anne L. M. Vlek, Corianne Meijer, Eva A. H. van Ravensbergen, Elisabeth G. W. Huijskens, Saara J. Vainio, Marc J. M. Bonten, Maaike S. M. van Mourik

**Affiliations:** 1 Department of Medical Microbiology and Infection Prevention, University Medical Centre Utrecht, P.O. Box 85500, 3508 GA Utrecht, The Netherlands; 2 Julius Centre for Health Sciences and Primary Care, University Medical Centre Utrecht, Utrecht, The Netherlands; 3 Department of Epidemiology and Surveillance, Centre for Infectious Diseases Control, National Institute for Public Health and the Environment, Bilthoven, The Netherlands; 4 Department of Medical Microbiology and Infection Prevention, Haaglanden Medical Centre, The Hague, the Netherlands; 5 Department of Medical Microbiology and Immunology, Diakonessenhuis Utrecht, Utrecht, The Netherlands; 6 Department of Medical Microbiology, Radboud Centre for Infectious Diseases, Radboud University Medical Centre, Nijmegen, The Netherlands; 7 Department of Infection Prevention and Control, Alrijne Zorggroep, Leiderdorp, The Netherlands; 8 Department of Medical Microbiology, Albert Schweitzer Hospital, Dordrecht, The Netherlands; 9 Department of Medical Microbiology, Immunology and Infection Prevention, St.Antonius Hospital, Nieuwegein, The Netherlands

**Keywords:** Inter-rater reliability, Surveillance, Infection prevention, Epidemiology, Colorectal surgery, Surgical site infection

## Abstract

**Background:**

Surveillance is the cornerstone of surgical site infection prevention programs. The validity of the data collection and awareness of vulnerability to inter-rater variation is crucial for correct interpretation and use of surveillance data. The aim of this study was to investigate the reliability and validity of surgical site infection (SSI) surveillance after colorectal surgery in the Netherlands.

**Methods:**

In this multicentre prospective observational study, seven Dutch hospitals performed SSI surveillance after colorectal surgeries performed in 2018 and/or 2019. When executing the surveillance, a local case assessment was performed to calculate the overall percentage agreement between raters within hospitals. Additionally, two case-vignette assessments were performed to estimate intra-rater and inter-rater reliability by calculating a weighted Cohen’s Kappa and Fleiss’ Kappa coefficient. To estimate the validity, answers of the two case-vignettes questionnaires were compared with the answers of an external medical panel.

**Results:**

1111 colorectal surgeries were included in this study with an overall SSI incidence of 8.8% (n = 98). From the local case assessment it was estimated that the overall percent agreement between raters within a hospital was good (mean 95%, range 90–100%). The Cohen’s Kappa estimated for the intra-rater reliability of case-vignette review varied from 0.73 to 1.00, indicating substantial to perfect agreement. The inter-rater reliability within hospitals showed more variation, with Kappa estimates ranging between 0.61 and 0.94. In total, 87.9% of the answers given by the raters were in accordance with the medical panel.

**Conclusions:**

This study showed that raters were consistent in their SSI-ascertainment (good reliability), but improvements can be made regarding the accuracy (moderate validity). Accuracy of surveillance may be improved by providing regular training, adapting definitions to reduce subjectivity, and by supporting surveillance through automation.

## Introduction

Surgical site infections (SSI) are one of the most common healthcare-associated infections (HAI) [1], and are associated with substantial morbidity and mortality, increased length of hospital stay and costs [2–6]. The highest SSI incidences are reported after colorectal surgeries, possibly due to the risk of (intra-operative) bacterial contamination and post-operative complications [7–9]. Worldwide, incidence rates range from 5 to 30% and are affected by several risk factors, including the type of surgery, age, sex, underlying health status, diabetes mellitus, blood transfusion, ostomy creation, prophylactic antibiotic use [10–12] and by the definition used to identify SSIs [4, 13].

Surveillance is an important component of prevention initiatives and most surveillance programs include colorectal surgeries [14]. Large variabilities in SSI rates between centres remain, even after correction for factors that increase the risk of SSIs. Previous studies reported significant variability in surveillance methodology and in inter-rater agreement, introducing uncertainty regarding whether observed differences in colorectal SSI rates reflect real differences in hospital performance [15–21].

For the purpose of comparing SSI rates between hospitals, accurate adherence to standardized surveillance protocols is required. Furthermore, case definitions should be unambiguous to avoid subjective interpretation. To reduce subjectivity the Dutch national surveillance network (PREZIES) has modified the case-definition on two criteria as compared to the definitions set out by the (European) Center of Disease Control and Prevention ((E)CDC) [22–25]. First, the diagnosis of an SSI made by a surgeon or attending physician only is not incorporated in the Dutch definitions. Second, in case of anastomotic leakage or bowel perforation, a deep or organ-space SSI can only be scored by purulent drainage from the deep incision, or when there is an abscess or other evidence of infection involving the deep soft tissues found on direct examination. A positive culture obtained from the (deep) tissue is not applicable in case of anastomotic leakage. Moreover, to increase standardization, the Dutch surveillance only includes primary resections of the large bowel and rectum, in contrast to the (E)CDC, who also allows biopsy procedures, incisions, colostomies or secondary resections.

Awareness of the correctness of applying the definition and vulnerability to inter-rater variation is crucial for correct interpretation and use of surveillance data. The aim of this study was to investigate the reliability and validity of SSI surveillance after colorectal surgery using the Dutch (PREZIES) SSI definitions and protocol. Secondary aims were to report the accuracy of determining anastomotic leakage and to provide insights in the SSI incidence and epidemiology in the Netherlands.

## Methods

### Study design

In this multicentre prospective observational study, seven Dutch hospitals (academic (tertiary referral university hospital) n = 2; teaching n = 3; general n = 2) collected surveillance data for occurrence of SSI after colorectal surgeries performed in 2018 and/or 2019, according to the Dutch PREZIES surveillance protocol [23, 25, 26]. Three hospitals had no prior experience in performing SSI surveillance after colorectal surgeries and four hospitals already performed this surveillance for more than five years as part of their quality program. Participation in SSI surveillance after colorectal surgery is voluntary, hence not all hospitals include this in their surveillance programme. When executing the surveillance, additionally intra- and inter-rater reliability and validity were determined by two case-vignette assessments and a local case assessment. Reliability refers to the consistency and reproducibility of SSI-ascertainment and was determined by three agreement measures: 1) the intra-rater reliability, reflecting the agreement within one single rater over time; 2) the inter-rater reliability, which is the agreement between two raters within one hospital; and 3) the overall inter-rater reliability between all 14 raters of seven hospitals [27, 28]. Validity refers to how accurately the surveillance definition is applied and was determined by the correctness of ascertainment compared to a medical panel as described in detail below. The Medical Ethical Committee of the University Medical Centre Utrecht approved this study and waived the requirement of informed consent (reference number 19–493/C). All data were processed in accordance with the General Data Protection Regulation. Hospitals were randomly assigned the letters A-G for reporting of the results.

### SSI surveillance after colorectal surgery

All hospitals included all primary colorectal resections of the large bowel and rectum performed in 2018 and/or 2019 in patients above the age of 1 year. Per hospital two raters, mostly ICPs, manually reviewed the electronic medical records for all included procedures retrospectively and classified procedures into three categories: (1) no SSI, (2) superficial SSI or (3) deep SSI or organ-space SSI within a follow-up period of 30 days post-surgery. SSIs were registered in their own hospital’s surveillance registration system. All identified SSIs and questionable cases were validated and discussed with each facility’s medical microbiologist or surgeon after completing the assessments which are described below.

### Case-vignette assessment

Case-vignettes were used to assess the validity, intra-rater and inter-rater reliability. Four medical doctors developed standardised case-vignettes in Dutch language, based on 20 patients selected from a previous study [29]. Each vignette described demographics, the medical history, type of surgical procedure and the postoperative course. An external medical panel of seven experts in the field of colorectal surgeries and surveillance classified the case-vignettes as a superficial SSI, deep SSI, or no SSI according to the Dutch SSI definition, and indicated presence or absence of anastomotic leakage. Their conclusion was considered the reference standard. Each rater who performed surveillance completed the case-vignettes individually through an online questionnaire. Three months later, the same vignettes were judged once more by the same raters, but presented in a different random order.

### Local case assessment

The reliability of surveillance data also depends on the ability to find the information necessary for case-ascertainment in the medical records. As this is not measured by the case-vignettes, we additionally performed a local case assessment: within each hospital, 25 consecutive colorectal surgeries included in surveillance were scored independently by the two raters, on separate digital personal forms. After sending the completed forms to the research team, raters discussed the results and entered the final decision into their hospital’s surveillance registration system.

### Training

Before starting the surveillance activities, a training session was organized to ensure the quality of the data collection and to practice SSI case-ascertainment. Thereby, before starting the reliability assessments, each ICP had to complete at least 20 inclusions for surveillance to assure familiarity with the surveillance procedure. In case of any questions, the research team was available to provide assistance.

### Statistical analyses

Descriptive statistics were generated to describe the surveillance period, number of inclusions and epidemiology. The number of SSIs per hospital were reported and displayed in funnel plots. The primary outcomes of this study were the reliability and validity of the surveillance. From the case-vignette assessments, the intra-rater and inter-rater reliability were analysed by calculating a weighted Cohen’s Kappa coefficient (κ). The scale used to interpret the κ estimates was as follows: ≤ 0, no agreement; 0.01–0.20, slight agreement; 0.21–0.40, fair agreement; 0.41–0.60, moderate agreement; 0.61–0.80, substantial agreement; 0.81–1.00, almost perfect agreement [27]. For the inter-rater reliability within a hospital, we used the second questionnaire round of the case-vignettes, to account for a possible learning curve over time. The overall inter-rater reliability among all 14 raters was estimated using a weighted Fleiss’ Kappa. For all Kappa’s, 95%-confidence intervals were estimated using bootstrapping methods (1000 repetitions). Inter-rater reliability was also measured from the local case assessment, from which the overall percentage agreement was calculated per hospital. Validity was determined by comparing the answers of the two case-vignettes questionnaires with the answers of the medical panel. The same comparison was performed to investigate the accuracy related to the determination of anastomotic leakage. Analyses were performed with R version 3.6.1 (R Foundation for Statistical Computing, Vienna, Austria) [30] with the use of packages *irr* [31] for inter-rater reliability and the *boot* [32] package for bootstrapping.

## Results

### Epidemiology

**Fig. 1:**
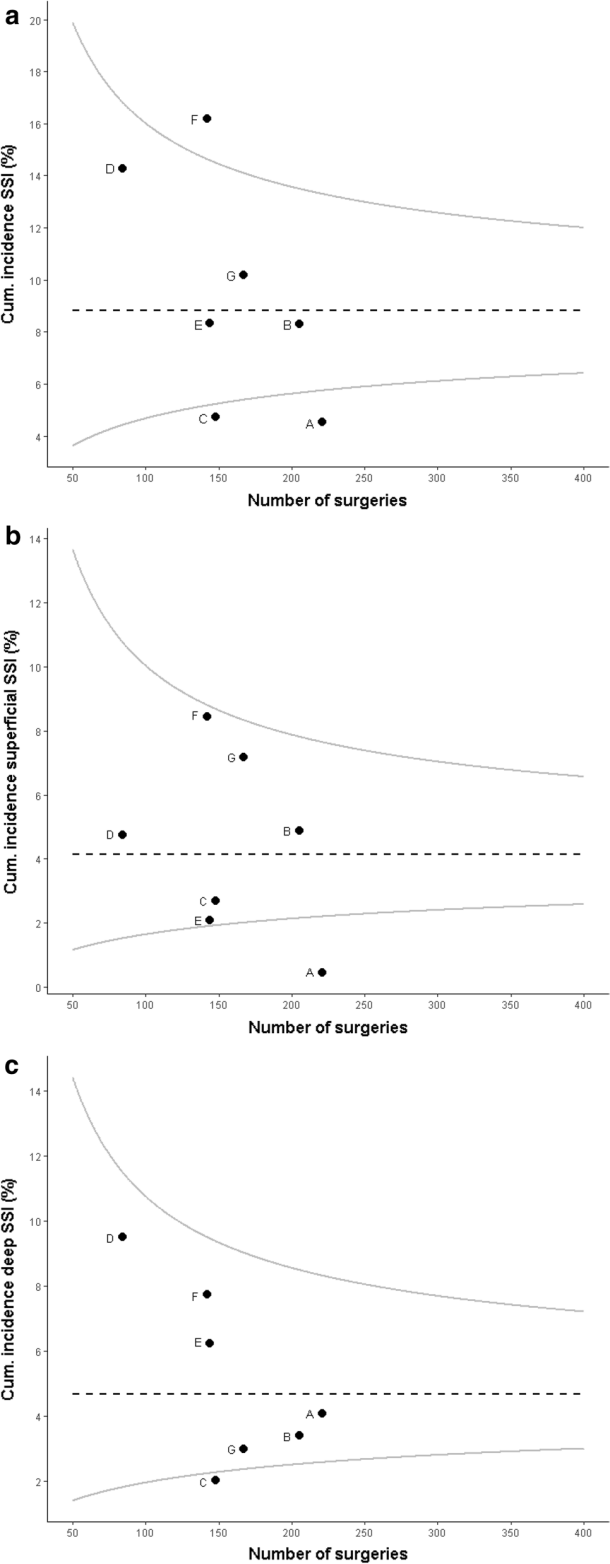
Overview of SSI incidence per hospital accounting for the number of surgical procedures. The black dotted line shows the mean incidence rate, the grey curved lines are the corresponding 95% confidence interval. **a** Overview of all SSIs per hospital. **b** Overview of superficial SSIs per hospital. **c** Overview of deep SSIs per hospital

1111 colorectal surgeries were included in the surveillance, in majority right-sided hemicolectomies (n = 445, 40.1%). The overall incidence of SSI was 8.8% (n = 98); 46.9% developed superficial SSI (n = 46) versus 53.1% deep SSI (n = 52). In 23 deep SSIs (44.2%) there was anastomotic leakage. Table 1 provides an overview of the cumulative incidence of SSIs per hospital and Fig. 1 displays the incidence of SSIs taking into account the number of surgical procedures. SSIs were observed more frequently in open surgeries than laparoscopic procedures, with the highest SSI incidence in open sigmoid colectomies (19.4%), followed by open left hemicolectomies, open right hemicolectomies and open low anterior resections (17.5%, 11.0% and 9.6% respectively). Other risk factors are shown in Table 2.

**Table 1:** Overview of colorectal surgeries and number of SSIs per participating hospital

	Type of hospital	Surveillance period	Number of colorectal surgeries (n)	Superficial SSI (n, %)	Deep SSI (n, %)	Total SSIs (n, %)
Hospital A	General	2019	221	1 (0.5%)	9 (4.1%)	10 (4.5%)
Hospital B	Teaching	2019	205	10 (4.9%)	7 (3.4%)	17 (8.3%)
Hospital C	General	2019	148	4 (2.7%)	3 (2.0%)	7 (4.7%)
Hospital D	Academic	2018–2019	84	4 (4.8%)	8 (9.5%)	12 (14.3%)
Hospital E*	Teaching	2019^a^	144	3 (2.1%)	9 (6.3%)	12 (8.3%)
Hospital F*	Teaching	2019^a^	142	12 (8.5%)	11 (7.7%)	23 (16.2%)
Hospital G*	Academic	2018-2019^a^	167	12 (7.2%)	5 (3.0%)	17 (10.2%)
Total			1111	46 (4.1%)	52 (4.7%)	98 (8.8%)

*SSI* surgical site infection, *n* number

^*^Hospitals that started surveillance for the purpose of this study

^a^January–June 2019

**Table 2:** Baseline characteristics and risk factors of patients who underwent a primary colorectal surgery

	No SSI (n = 1013)	Superficial SSI (n = 46)	Deep SSI (n = 52)
Sex (n, (%))			
Male	506 (50.0)	29 (63.0)	31 (59.6)
Female	507 (50.0)	17 (37.0)	21 (40.4)
Age in years (mean, (SD))	65.7 (13.7)	61.8 (15.0)	63.2 (15.4)
*Pre-operative risk factors*			
BMI (mean, (SD))	26.1 (4.6)	27.0 (4.8)	27.6 (7.0)
Missing (n, (%))	29 (2.9)	2 (4.3)	2 (3.8)
ASA grade (n, (%))			
Grade I	94 (9.3)	5 (10.9)	3 (5.8)
Grade II	542 (53.5)	20 (43.5)	24 (46.2)
Grade III	289 (28.5)	12 (26.1)	17 (32.7)
Grade IV	43 (4.2)	5 (10.9)	2 (3.8)
Grade V	7 (0.7)	-	-
Missing (n, (%))	38 (3.8)	4 (8.6)	6 (11.5)
*Procedure-related risk factors*			
Type of surgery (n, (%))			
Right hemicolectomy, closed procedure	285 (28.1)	9 (19.6)	6 (11.5)
Right hemicolectomy, open procedure	129 (12.7)	6 (13.0)	10 (19.3)
Left hemicolectomy, closed procedure	72 (7.1)	1 (2.2)	5 (9.6)
Left hemicolectomy, open procedure	33 (3.3)	3 (6.5)	4 (7.7)
Sigmoid colectomy closed procedure	171 (16.9)	2 (4.3)	5 (9.6)
Sigmoid colectomy open procedure	108 (10.7)	17 (37.0)	9 (17.3)
Low anterior colectomy, closed procedure	168 (16.6)	4 (8.7)	12 (23.1)
Low anterior colectomy, open procedure	47 (4.6)	4 (8.7)	1 (1.9)
Surgical approach (n, (%))			
Closed	696 (68.7)	16 (34.8)	28 (53.8)
Open	317 (31.3)	30 (65.2)	24 (46.2)
Duration of surgery in minutes (median, (IQR))^a^	132 (68)	143 (64)	137 (56)
Missing (n, (%))	11 (1.1)	-	-
Emergency (n, (%))^b^			
Yes	124 (18.8)	13 (48.1)	12 (40.0)
No	528 (80.1)	14 (51.9)	18 (60.0)
Missing (n, (%))	7 (1.1)	-	-
Wound class (n, (%))^*c*^			
Clean-contaminated (class 2)	724 (81.0)	20 (58.8)	26 (63.4)
Contaminated (class 3)	104 (11.6)	2 (5.9)	7 (17.1)
Dirty-infected (class 4)	65 (7.3)	11 (32.4)	8 (19.5)
Missing (n, (%))	1 (0.1)	1 (2.9)	-
Malignancy (n, (%))			
Yes	695 (68.6)	24 (52.2)	33 (63.5)
No	243 (24.0)	20 (43.5)	16 (30.8)
Missing (n, (%))	75 (7.4)	2 (4.3)	3 (5.8)
Stoma (n, (%))			
Yes	233 (23.0)	28 (60.9)	22 (42.3)
No	780 (77.0)	18 (39.1)	30 (57.7)
*Post-operative risk factors*			
30-day mortality (n, (%)) ^d^			
Yes	28 (3.8)	1 (3.2)	4 (10.5)
No	703 (96.2)	30 (96.8)	34 (89.5)
ICU admission (n, (%)) ^e^			
Yes	162 (24.6)	11 (40.7)	16 (53.3)
No	497 (75.4)	16 (59.3)	14 (46.7)
*Microbiology*			
Microorganism (n,(%))			
No microorganism identified or no culture taken	NA	28 (60.9)	15 (28.8)
Positive culture ^f^	NA	18 (39.1)	37 (71.2)
Escherichia coli		6 (25.0)	20 (31.3)
Enterococcus faecalis		2 (8.3)	7 (10.9)
Enterococcus faecium		3 (12.5)	6 (9.3)
Pseudomonas aeruginosa		5 (20.8)	6 (9.3)
Klebsiella pneumonia		1 (4.2)	4 (6.3)
Staphylococcus aureus		2 (8.3)	0 (0.0)
Other		5 (20.9)	21 (32.9)

SSI, surgical site infection; n, number; SD, standard deviation; BMI, body mass index; ASA, American Society of Anaesthesiologists Physical Status; IQR, Interquartile range; ICU, Intensive Care Unit; NA, not applicable

^a^Not available for hospital F

^b^Not available for hospital D, E and G, so percentage was calculated without these hospitals

^c^Not available for hospital F, so percentage was calculated without this hospital

^d^Not available for hospital E and G, so percentage was calculated excluding these hospitals

^e^Not available for hospital D, E and G, so percentage was calculated excluding these hospitals

^f^Percentage was calculated relative to the total number of cultured microorganisms

### Reliability and validity

All 14 raters completed the two rounds of online questionnaire with case-vignettes. Of those, two had less than one year of experience with HAI surveillance, six had 2–5 years of experience, five persons 6–15 years and one more than 25 years. The estimated Cohen’s Kappa for agreement within a rater (intra-rater reliability) calculated from the case-vignette assessment varied from 0.73 to 1.00, indicating substantial to perfect agreement (Table 3). The inter-rater reliability within hospitals showed more variation, with lowest estimates reported for hospital A (κ = 0.61, 95%-CI 0.23–0.83) and the highest in hospital C (κ = 0.94, 95%-CI 0.75–1.00). The overall inter-rater agreement of all 14 raters in the second round case-vignettes was 0.72 (95%-CI 0.59–0.83). From the local case assessment it was estimated that the overall percent agreement between raters within a hospital was almost perfect (mean = 95%, range 90–100%). Regarding the accuracy of determining SSIs correctly, 87.9% (range 70%-95%) of the answers given by the raters were in accordance with the medical panel: 3 raters had similar SSI rates compared to the medical panel, five raters underestimated the number of SSIs, four had higher SSI rates because of incorrect ascertainment and there were two raters who had overestimated SSI in the first round, and an underestimation in the second round. Presence of anastomotic leakage was accurately scored in the vignettes where it was present, however misclassified in cases where anastomotic leakage was absent (Table 3).

**Table 3:** Intra-rater-, Inter-rater reliability and accuracy measured by two questionnaire rounds of 20 case vignettes each

		Years of working experience in infectious disease surveillance	Intra-rater reliability (κ, 95%-CI)	Inter-rater reliability per hospital (κ, 95%-CI)^#^	Accuracy (%, First round/Second round)	Accuracy in determination of presence of anastomotic leakage, n = 4. (%, First round/Second round)	Accuracy in determination of absence of anastomotic leakage, n = 16 (%, First round/Second round)
Hospital A	Rater 1	4–5	0.78 (0.46–1.00)	0.61 (0.23–0.83)	95/85	75/100	93/87
Hospital A	Rater 2	2–3	0.95 (0.74–1.00)		85/80	100/75	93/93
Hospital B	Rater 1	11–15	0.83 (0.49–0.99)	0.72 (0.42–1.00)	80/85	75/100	93/93
Hospital B	Rater 2	6–10	0.73 (0.44–1.00)		95/90	100/100	93/93
Hospital C	Rater 1	11–15	1.00 (1.00–1.00)	0.94 (0.75–1.00)	90/90	75/75	93/93
Hospital C	Rater 2	11–15	0.94 (0.76–1.00)		90/95	75/75	93/93
Hospital D	Rater 1	0–1	0.75 (0.47–1.00)	0.69 (0.36–0.92)	90/85	100/100	93/87
Hospital D	Rater 2	4–5	0.89 (0.72–1.00)		90/95	100/100	93/87
Hospital E*	Rater 1	2–3	0.89 (0.59–1.00)	0.65 (0.38–0.92)	80/80	100/100	93/93
Hospital E*	Rater 2	4–5	0.73 (0.46–1.00)		85/70	100/100	93/81
Hospital F*	Rater 1	2–3	0.79 (0.57–1.00)	0.69 (0.34–0.92)	90/90	100/100	87/81
Hospital F*	Rater 2	11–15	0.89 (0.59–1.00)		90/90	100/100	87/87
Hospital G*	Rater 1	0–1	0.79 (0.55–1.00)	0.84 (0.61–1.00)	90/90	100/100	87/93
Hospital G*	Rater 2	> 25	0.94 (0.75–1.00)		95/90	100/100	93/93

κ, Cohen’s Kappa coefficient; 95% CI, 95% confidence interval, n, number

^*^Hospitals that started surveillance for the purpose of this study

^#^Inter-rater reliability was calculated from the second round questionnaire case vignettes

## Discussion

In this study we observed good reliability of SSI surveillance after colorectal surgeries in seven Dutch hospitals. Based on the case-vignette assessment, the intra-rater reliability was estimated substantial to perfect (κ = 0.71–1.00) and the inter-rater agreement within hospitals was substantial, but varied between hospitals (κ = 0.61–0.94). The local case assessment showed 95% agreement within hospitals. Despite the fact that individual raters were consistent in their scoring, validity was moderate: in 12.1% (range 5%-30%) the case-ascertainment was not correct as compared to the conclusions of the medical panel. The SSI rate determined by surveillance would therefore be under-or overestimated.

To the best of our knowledge, there is only one other study assessing the inter-rater reliability explicitly for SSI after colorectal surgeries. Hedrick et al. [18] concluded from their results that SSIs could not reliable be assigned and reproduced: they demonstrated large variation in SSI incidence between raters with only modest inter-rater reliability (i.e. κ = 0.64). They therefore opt for alternative definitions such as the ASEPSIS score [33]. In the present study similar estimates for inter-reliability were found in 2 out of 7 hospitals (κ = 0.61 in hospital A and κ = 0.65 in hospital E), for the other five hospital we found estimates above 0.69. The higher reliability estimates found in the present study may be explained by several factors. First, the definitions and method used in the Netherlands aim to be more objective: a previous study has shown that surgeon's diagnosis – not included the Dutch definition– lead to biased results [34, 35]. Another factor that may influence reliability is the years of surveillance experience of the raters and their ability to find information in the electronic health records needed for case-ascertainment [36]. From Table 3 it seems that more experienced raters produce more consistent results. However, the design of this study did not allow to investigate this type of causal relationships.

The reliability estimates of this study show that SSIs after colorectal surgery are an appropriate measure to use for surveillance: the same result can be consistently achieved, making them reproducible and suitable for monitoring trends and detecting changes in SSI rates within a hospital. However, at this moment, using SSI incidence as a quality measure for benchmarking may be hampered because of three reasons. First, we found that on average 12.1% of patients in the case-vignettes were misclassified: one rater misclassified 6 out of 20 vignettes while another had only one misclassification. This will lead to unreliable comparisons of SSI rates, although in practice difficult cases may be discussed in a team hence improving accuracy. As superficial SSIs rely on more subjective criteria, focusing on deep SSI may improve accuracy and comparability. Additionally, we observed that anastomotic leakage was too often assigned while it was actually absent. This may lead to an underestimation as these cases cannot be scored by a positive culture anymore according to the Dutch definition (as explained in the introduction). Second, Kao et al. [16] and Lawson et al. [15] investigated whether SSI surveillance after colorectal surgeries has good ability to differentiate high and low quality performance (i.e. the statistical reliability of SSIs). They both concluded that the measure can only be used as hospital quality measure when an adequate number of cases have been reported, which can be challenging for some hospitals as shown in Table 1. Third, another challenge in using SSI rates for interhospital comparisons is the lack of a sufficient method for risk adjustment. To obtain valid SSI comparisons, you have to correct for differences in the surveillance population and their risk factors. However, to date no method has been proven generalizable and appropriate [12, 37]. The points raised above show that the overall SSI incidence of 8.8% in this study is difficult to compare to others. Overall, the SSI incidence was lower compared to other studies, but in line with numbers previously reported to the Dutch national surveillance network [13, 38, 39].

When SSIs after colorectal surgery are used for monitoring and perhaps benchmarking, continuous training of raters is required to assure correct use and alignment of surveillance definitions and methodology. Reliability and validity of surveillance may be improved by automatization methods as they can help to support case-finding [40–42]. Furthermore, hospitals should perform a certain number of colorectal surgeries to generate representative estimates of performance. If there is no appropriate case-mix correction, comparisons should be made with caution, preferably between similar types of hospitals with comparable patient groups.

### Strengths and limitations

This study was performed within multiple Dutch centres, including different types of hospitals. The 14 raters in this study were well-trained according to standardized methods to minimalize differences possibly caused by years of surveillance experiences between hospitals. Unfortunately, this design was not suitable for explaining which factors enhance SSI-ascertainment or will improve reliability and validity estimates. Second, we aimed to produce Cohen’s Kappa coefficients from the local case assessment as well, however it appeared that there was too little variation in outcomes and number of cases hindering this calculation.

## Conclusion

Awareness of the validity of surveillance and vulnerability to inter-rater variation is crucial for correct interpretation and use of surveillance data. This study showed that raters were consistent in their SSI-ascertainment, but improvements can be made regarding the accuracy. Hence, SSI surveillance results for colorectal surgery are reproducible and thus suitable for monitoring trends, but not necessarily correct and therefore less adequate for benchmarking. Based on prior literature, accuracy of surveillance may be improved by providing regular training, adapting definitions to reduce subjectivity, and by supporting case-finding by automation.

## Data Availability

The datasets used and/or analyzed during the current study are available from the corresponding author on reasonable request.

## References

[1] Magill SS, Edwards JR, Bamberg W, Beldavs ZG, Dumyati G, Kainer MA (2014). Multistate point-prevalence survey of health care-associated infections. N Engl J Med.

[2] Koek MBG, van der Kooi TII, Stigter FCA, de Boer PT, de Gier B, Hopmans TEM (2019). Burden of surgical site infections in the Netherlands: cost analyses and disability-adjusted life years. J Hosp Infect.

[3] Kirkland KB, Briggs JP, Trivette SL, Wilkinson WE, Sexton DJ (1999). The impact of surgical-site infections in the 1990s: attributable mortality, excess length of hospitalization, and extra costs. Infect Control Hosp Epidemiol.

[4] Tanner J, Khan D, Aplin C, Ball J, Thomas M, Bankart J (2009). Post-discharge surveillance to identify colorectal surgical site infection rates and related costs. J Hosp Infect.

[5] Shaw E, Gomila A, Piriz M, Perez R, Cuquet J, Vazquez A (2018). Multistate modelling to estimate excess length of stay and risk of death associated with organ/space infection after elective colorectal surgery. J Hosp Infect.

[6] Mahmoud NN, Turpin RS, Yang G, Saunders WB (2009). Impact of surgical site infections on length of stay and costs in selected colorectal procedures. Surg Infect (Larchmt).

[7] Claesson BEB, Holmlund DEW (1988). Predictors of intraoperative bacterial contamination and postoperative infection in elective colorectal surgery. J Hosp Infect.

[8] Hagihara M, Suwa M, Muramatsu Y, Kato Y, Yamagishi Y, Mikamo H (2012). Preventing surgical-site infections after colorectal surgery. J Infect Chemother.

[9] Shanahan F (2002). The host-microbe interface within the gut. Best Pract Res Clin Gastroenterol.

[10] Tserenpuntsag B, Haley V, Van Antwerpen C, Doughty D, Gase KA, Hazamy PA (2014). Surgical site infection risk factors identified for patients undergoing colon procedures, New York State 2009–2010. Infect Control Hosp Epidemiol.

[11] Tang R, Chen HH, Wang YL, Changchien CR, Chen JS, Hsu KC (2001). Risk factors for surgical site infection after elective resection of the colon and rectum: a single-center prospective study of 2,809 consecutive patients. Ann Surg.

[12] Grant R, Aupee M, Buchs NC, Cooper K, Eisenring MC, Lamagni T (2019). Performance of surgical site infection risk prediction models in colorectal surgery: external validity assessment from three European national surveillance networks. Infect Control Hosp Epidemiol.

[13] Limón E, Shaw E, Badia JM, Piriz M, Escofet R, Gudiol F (2014). Post-discharge surgical site infections after uncomplicated elective colorectal surgery: impact and risk factors. The experience of the VINCat Program. J Hosp Infect.

[14] Abbas M, de Kraker MEA, Aghayev E, Astagneau P, Aupee M, Behnke M (2019). Impact of participation in a surgical site infection surveillance network: results from a large international cohort study. J Hosp Infect.

[15] Lawson EH, Ko CY, Adams JL, Chow WB, Hall BL (2013). Reliability of evaluating hospital quality by colorectal surgical site infection type. Ann Surg.

[16] Kao LS, Ghaferi AA, Ko CY, Dimick JB (2011). Reliability of superficial surgical site infections as a hospital quality measure. J Am Coll Surg.

[17] Degrate L, Garancini M, Misani M, Poli S, Nobili C, Romano F (2011). Right colon, left colon, and rectal surgeries are not similar for surgical site infection development. Analysis of 277 elective and urgent colorectal resections. Int J Colorectal Dis.

[18] Hedrick TL, Sawyer RG, Hennessy SA, Turrentine FE, Friel CM (2014). Can we define surgical site infection accurately in colorectal surgery?. Surg Infect (Larchmt).

[19] Reese SM, Knepper BC, Price CS, Young HL (2015). An evaluation of surgical site infection surveillance methods for colon surgery and hysterectomy in Colorado hospitals. Infect Control Hosp Epidemiol.

[20] Ming DY, Chen LF, Miller BA, Anderson DJ (2012). The impact of depth of infection and postdischarge surveillance on rate of surgical-site infections in a network of community hospitals. Infect Control Hosp Epidemiol.

[21] Pop-Vicas A, Stern R, Osman F, Safdar N (2020). Variability in infection surveillance methods and impact on surgical site infection rates. Am J Infect Control.

[22] Surveillance of surgical site infections and prevention indicators in European hospitals - HAI-Net SSI protocol, version 2.2. In: ECDC, editor. Stockholm: European Centre for Disease Prevention and Control; 2017.

[23] PREZIES. Case Definitions SSIs Bilthoven: National Institute for Public Health and the Environment; 2020. https://www.rivm.nl/documenten/case-definitions-ssis. Accessed 22 May 2021.

[24] National Healthcare Safety Network (NHSN): patient safety component manual. Atlanta: CDC; 2019.

[25] Verberk JDM, Meijs AP, Vos MC, Schreurs LMA, Geerlings SE, de Greeff SC (2017). Contribution of prior, multiple-, and repetitive surgeries to the risk of surgical site infections in the Netherlands. Infect Control Hosp Epidemiol.

[26] PREZIES. Protocol en dataspecificaties, module POWI Bilthoven: National Institute for Public Health and the Environment; 2019. https://www.rivm.nl/sites/default/files/2018-11/Protocol%20en%20DS%20POWI_2019_v1.0_DEF.pdf. Accessed 10 Jan 2020.

[27] McHugh ML (2012). Interrater reliability: the kappa statistic. Biochem Med (Zagreb).

[28] Hallgren KA (2012). Computing inter-rater reliability for observational data: an overview and tutorial. Tutor Quant Methods Psychol.

[29] Mulder T, Kluytmans-van den Bergh MFQ, de Smet A, van’t Veer NE, Roos D, Nikolakopoulos S (2018). Prevention of severe infectious complications after colorectal surgery using preoperative orally administered antibiotic prophylaxis (PreCaution): study protocol for a randomized controlled trial. Trials.

[30] R Core Team. R: A language and environment for statistical computing. Vienna: R Foundation for Statistical Computing; 2020. https://www.R-project.org. Accessed.

[31] Gamer M, Lemon J, Fellows I, Singh P. irr: various coefficients of interrater reliability and agreement 2019. Version 0.84.1:[https://cran.r-project.org/web/packages/irr/index.html. Accessed 10 Dec 2020.

[32] Canty A, Ripley B. boot: Bootstrap Functions (Originally by Angelo Canty for S) 2020. Version 1.3–25:[https://cran.r-project.org/web/packages/boot/index.html. Accessed 21 Nov 2020.

[33] Wilson AP, Treasure T, Sturridge MF, Grüneberg RN (1986). A scoring method (ASEPSIS) for postoperative wound infections for use in clinical trials of antibiotic prophylaxis. Lancet.

[34] Taylor G, McKenzie M, Kirkland T, Wiens R (1990). Effect of surgeon's diagnosis on surgical wound infection rates. Am J Infect Control.

[35] Wilson AP, Gibbons C, Reeves BC, Hodgson B, Liu M, Plummer D (2004). Surgical wound infection as a performance indicator: agreement of common definitions of wound infection in 4773 patients. BMJ.

[36] Ehrenkranz NJ, Shultz JM, Richter EL (1995). Recorded criteria as a "gold standard" for sensitivity and specificity estimates of surveillance of nosocomial infection: a novel method to measure job performance. Infect Control Hosp Epidemiol.

[37] Bergquist JR, Thiels CA, Etzioni DA, Habermann EB, Cima RR (2016). Failure of colorectal surgical site infection predictive models applied to an independent dataset: do they add value or just confusion?. J Am Coll Surg.

[38] PREZIES. Referentiecijfers 2014–2018: Postoperatieve Wondinfecties: National Institute for Public Health and the Environment; 2019. https://www.rivm.nl/documenten/referentiecijfers-powi-2018. Accessed 11 Nov 2020.

[39] Hübner M, Diana M, Zanetti G, Eisenring M-C, Demartines N, Troillet N (2011). Surgical site infections in colon surgery: the patient, the procedure, the hospital, and the surgeon. Arch Surg.

[40] Rusk A, Bush K, Brandt M, Smith C, Howatt A, Chow B (2016). Improving surveillance for surgical site infections following total hip and knee arthroplasty using diagnosis and procedure codes in a provincial surveillance network. Infect Control Hosp Epidemiol.

[41] Verberk JDM, van Rooden SM, Koek MBG, Hetem DJ, Smilde AE, Bril WS (2020). Validation of an algorithm for semiautomated surveillance to detect deep surgical site infections after primary total hip or knee arthroplasty—a multicenter study. Infect Control Hosp Epidemiol.

[42] Trick WE (2013). Decision making during healthcare-associated infection surveillance: a rationale for automation. Clin Infect Dis.

